# Characterization of human Spartan/C1orf124, an ubiquitin-PCNA interacting regulator of DNA damage tolerance

**DOI:** 10.1093/nar/gks850

**Published:** 2012-09-16

**Authors:** Szilvia Juhasz, David Balogh, Ildiko Hajdu, Peter Burkovics, Mark A. Villamil, Zhihao Zhuang, Lajos Haracska

**Affiliations:** ^1^Institute of Genetics, Biological Research Centre, Hungarian Academy of Sciences, Szeged 6726, Hungary, ^2^Department of Genetics, Howard Hughes Medical Institute, Division of Genetics, Brigham and Women's Hospital, Harvard University Medical School, Boston, MA 02115 and ^3^Department of Chemistry and Biochemistry, University of Delaware, Newark, DE 19716, USA

## Abstract

Unrepaired DNA damage may arrest ongoing replication forks, potentially resulting in fork collapse, increased mutagenesis and genomic instability. Replication through DNA lesions depends on mono- and polyubiquitylation of proliferating cell nuclear antigen (PCNA), which enable translesion synthesis (TLS) and template switching, respectively. A proper replication fork rescue is ensured by the dynamic ubiquitylation and deubiquitylation of PCNA; however, as yet, little is known about its regulation. Here, we show that human Spartan/C1orf124 protein provides a higher cellular level of ubiquitylated-PCNA by which it regulates the choice of DNA damage tolerance pathways. We find that Spartan is recruited to sites of replication stress, a process that depends on its PCNA- and ubiquitin-interacting domains and the RAD18 PCNA ubiquitin ligase. Preferential association of Spartan with ubiquitin-modified PCNA protects against PCNA deubiquitylation by ubiquitin-specific protease 1 and facilitates the access of a TLS polymerase to the replication fork. In concert, depletion of Spartan leads to increased sensitivity to DNA damaging agents and causes elevated levels of sister chromatid exchanges. We propose that Spartan promotes genomic stability by regulating the choice of rescue of stalled replication fork, whose mechanism includes its interaction with ubiquitin-conjugated PCNA and protection against PCNA deubiquitylation.

## INTRODUCTION

The genome is constantly under assault from chemical agents and radiation, which is especially deleterious in S-phase, when replication forks may stall upon encountering unrepaired DNA lesions leading to double-strand break formation and DNA rearrangements (1,2). The rescue of stalled replication forks is governed by the Rad6–Rad18 ubiquitin ligase complex-dependent monoubiquitylation of proliferating cell nuclear antigen (PCNA), the DNA polymerase processivity factor, that facilitates translesion synthesis (TLS) providing direct nucleotide incorporation opposite DNA lesions (3–6). In addition, monoubiquitylated PCNA can be polyubiquitylated by K63 ubiquitin linkage leading to template switching, in which copying from the undamaged newly synthesized sister strand can lead to error-free DNA damage bypass (7–13). In the absence of PCNA ubiquitylation, recombination using the sister chromatid can facilitate an alternative means for fork rescue (14–18). However, it has been suggested that reviving replication forks by recombination is disadvantageous for cells, probably because it can lead to gross chromosomal rearrangements. To keep recombination under control, SUMO modification of PCNA plays an important role by recruiting the Rad51 displacing helicase and channeling fork rescue to the Rad6–Rad18-dependent damage tolerance pathway (19–22).

Although the operation of the Rad6–Rad18-dependent damage bypass pathway is considered sufficient to suppress gross chromosomal rearrangements, untimely DNA synthesis by low-fidelity TLS polymerases could also lead to genome instability by increasing the rate of point mutations. Recent studies have indicated that for providing timely and only limited access of a TLS polymerase to the 3′-primer-end PCNA ubiquitylation and deubiquitylation might play equally important role. Although PCNA ubiquitylation promotes the access of a TLS polymerase to the replication fork, PCNA deubiquitylation can initiate the displacement of a TLS polymerase and provide the restoration of the normal DNA synthesis by the high-fidelity polymerase after replication through the lesion (23–27). Moreover, PCNA deubiquitylation can inhibit the untimely access of low-fidelity TLS polymerases and other players to the replication fork (24,28).

One level of the control of the lifetime of ubiquitylated-PCNA is provided by ubiquitin-specific protease 1 (USP1) existing in complex with the USP1-associated factor 1 (UAF1) stimulatory subunit, which can deubiquitylate PCNA (24,29–31). USP1-dependent PCNA deubiquitylation is regulated, on one hand, by UV irradiation-dependent inactivation of USP1 through an autocleavage event and, on the other hand, by the PCNA-interacting ELG1 protein that is essential for recruiting USP1-UAF1 to the stalled fork (26,32). However, understanding the regulation of the dynamism of PCNA ubiquitylation and deubiquitylation and its consequences on protein recruitments to the stalled replication fork and on the choice of DNA damage tolerance (DDT) pathways are still in a rudimentary stage.

Recently, Spartan/C1orf124 has been identified in parallel with our study and shown to be a reader of PCNA ubiquitylation and a regulator of UV-induced DNA damage response (33). However, no experimental insight into how Spartan affects the cellular level of ubiquitin-PCNA has been provided, and its influence on alternative DDT pathways remained unclear.

We now report the further characterization of human Spartan/C1orf124 and provide evidence for its regulatory role in DDT. Notably, we suggest that by preferential binding to ubiquitylated PCNA, Spartan protects against PCNA deubiquitylation by USP1 and facilitates the access of a TLS polymerase to the replication fork. We discuss the possibility that Spartan promotes genomic stability by preventing recombination and channeling fork rescue to PCNA ubiquitylation-dependent pathways.

## MATERIALS AND METHODS

### Generating Spartan DNA constructs

*SPARTAN* (C1orf124) clone was purchased from *ImaGenes* (cat.: DKFZp547N043Q), and we renamed it as pIL1767. Spartan cDNA was sequence verified and cloned into pENTR2B resulting in plasmid pIL1783. Point mutants were generated using pIL1783 by the QuickChange site-directed mutagenesis kit (*Stratagene*) resulting in UBZ mutant C459S Spartan (pIL2026), PIP mutant YF331,332AA Spartan (pIL2115), SprT mutant HE111,112AA Spartan (pIL2234) and UBZ, PIP double mutant C459S,YF331,332AA Spartan (pIL2210). The wild-type (WT), C459S and HE111,112AA siRNA-resistant Spartan constructs were generated using pIL1783, pIL2026 and pIL2234 and the following oligonucleotides O2662: (5′-CTA AGC AAC TAC TTT CCG CGA GTA TCA TTT GCC AAC C-3′) and O2663 (5′-GGT TGG CAA ATG ATA CTC GCG GAA AGT AGT TGC TTA G-3′) resulting in pIL2235, pIL2236, pIL2237, respectively. The YF331,332AA mutant siRNA-resistant Spartan was generated using pIL2115 and the following oligonucleotides O2685: (5′-CCA AAA TGT TCT AAG CAA CGC CGC TCC GCG AGT ATC ATT TGC C-3′) and O2686: (5′-GGC AAA TGA TAC TCG CGG AGC GGC GTT GCT TAG AAC ATT TTG G-3′) resulting in pIL2238. The C459S Y331A/F332A double siRNA-resistant mutant was generated by cloning the mutant fragment of pIL2236 to the plasmid pIL2238, resulting pIL2239 plasmid.

For localization studies, the WT, SPRT domain, UBZ domain, PIP box and double UBZ PIP box mutant Spartan cDNAs were cloned in fusion with N-terminal FLAG-tag into plasmid pRK2F resulting in plasmids pIL2240, pIL2241, pIL2242, pIL2243 and pIL2244, respectively. The WT Spartan was also cloned in fusion with EGFP-tag into plasmid pEGFP (pIL1785).

For expressing Spartan protein for protein purification, the WT, UBZ, PIP and double UBZ PIP domain mutant Spartan cDNAs were cloned in N-terminal fusion with glutathione S-transferase (GST) followed by a FLAG-tag under the control of the *Saccharomyces cerevisiae* galactose-inducible phosphoglycerate promoter resulting in plasmids pIL2116, pIL2117, pIL2119 and pIL2218, respectively.

For complementation assay, we cloned the WT, SprT-, UBZ-, PIP- and double PIP-UBZ domain mutant shRNA-resistant Spartan cDNAs in fusion with N-terminal FLAG-tag into plasmid pRK2F, which resulted in plasmids pIL2246, pIL2247, pIL2248, pIL2249 and pIL2250, respectively.

### Protein purifications

The WT and mutant GST-Flag-Spartan proteins were expressed in parallel in the protease-deficient BJ5464 yeast strain followed by purification on glutathione-Sepharose beads. The proteins were eluted from the beads either by 20 mM reduced glutathione resulting in GST-FLAG-Spartan or by PreScission protease resulting in FLAG-Spartan.

The human USP1-UAF1 was expressed in Sf9 cells and purified on Ni-NTA agarose resin as described previously (30,31). The concentration of purified proteins was measured using Bradford assay.

The human Rad6–Rad18, Mms2-Ubc13, RFC, UBA1, ubiquitin and HLTF proteins were purified, and the *in vitro* PCNA mono- and polyubiquitilation reactions were carried out as described previously (12). For obtaining highly purified mono- and K63 polyubiquitin-PCNA, the DNA from the ubiquitylation reaction was first digested by DNaseI followed by protein purification on a Q-sepharose and a subsequent Ni-NTA agarose chromatography whose fractions were analyzed by immunoblotting using anti-PCNA antibody.

### *In vitro* GST pull-down interaction assays of Spartan

Purified GST and GST-ubiquitin (3–3 μg) along with WT and mutant FLAG-Spartan proteins (1 μg), or WT and mutant GST-FLAG-Spartan (3–3 μg) along with PCNA, monoubiquitin-PCNA or polyubiquitin-PCNA (1 µg each) were incubated with glutathione-sepharose beads (40 μl) for 4 h at 4°C in buffer I (40 mM Tris HCl pH 7.5, 85 mM NaCl, 0.1 mM DTT, 10% glycerol, 0.01% NP40). Beads were washed three times with buffer I, and bound proteins were eluted in buffer I containing 20 mM reduced glutathione. Eluted fractions were analyzed by immunoblotting as indicated.

### *In vitro* PCNA deubiquitylation assay

Purified mono- and polyubiquitin PCNA (150 nM) was incubated with increasing concentration of USP1-UAF1 (0–125 nM) as indicated in buffer D (50 mM HEPES pH. 7.5, 5 mM MgCl_2_, 0.1 mg/ml BSA, 1 mM DTT) for 45 min at 37°C in the absence or presence of Spartan (150 nM). Reaction products were analyzed by immunoblotting with anti-PCNA antibody.

### Cell cultures and cellular protein localization studies

HeLa, HCT116 and HEK293FT were grown in Dulbecco’s modified Eagle’s medium (Sigma) supplemented with 10% FCS (Sigma) at 37°C. Transfections were carried out using Lipofectamine 2000 transfection reagent (Invitrogen) according to the instruction of the manufacturer. After treatment with UV (20 J/m^2^) or MMS (0.01%), cells were incubated for 3 h and immunostained where indicated.

For cellular localizations of Spartan and Polη, cells were immunostained (34) using anti-FLAG antibody (Sigma, cat. numb.: F7425) diluted 1:200 and FITC-conjugated anti-rabbit antibody (DAKO) diluted 1:1000 or anti-PCNA antibody (Santa Cruz, sc-56) diluted 1:200 and Cy3-conjugated anti-mouse antibody (Sigma, cat. numb.: C2181) diluted 1:1000. Samples were mounted in 25% glycerol in phosphate-buffered saline (PBS) containing 1 μg/ml DAPI followed by microscopy using an Olympus FV1000 confocal laser scanning microscope and a Leica confocal LSM.

For the BrdU procedure, the cells were labeled with BrdU (25 µM in DMEM) for 30 min at 37°C in a CO_2_ incubator. After washing, cells were fixed with 3% paraformaldehyde for 10 min at room temperature and denaturated by 2.5 M HCl for 30 min. Next, cells were treated with 0.5% Triton X-100 solution in PBS before blocking with 3% BSA in 0.1% Triton X-100 in PBS. BrdU was detected by anti-BrdU (Ab-direct serotec, cat. numb.: 170107) diluted 1:300 and anti-rat Alexa Fluor 488 (Invitrogen, Lot: 421559) diluted 1:1000. FLAG-Spartan was stained using anti-FLAG antibody (Sigma, M2 clone) diluted 1:200 and Cy3 conjugated anti-mouse antibody (Sigma, cat. numb.: C2181) diluted 1:1000.

### Laser microirradiation-induced DNA damage and immunofluorescence microscopy

Ultraviolet-laser-induced damage was generated as described previously (35). Cells were presensitized with 10 μM BrdU for 24 h to ultraviolet-A laser and microirradiated. After damage, cells were allowed to recover for 20 min at 37 °C and then fixed in 3.7% formaldehyde for 10 min at room temperature. Fixed cells were washed twice with PBS, permeabilized in 0.5% NP-40 for 5 min, washed twice with PBS and blocked with PTB (0.1% (w/v) NP-40, 1% BSA in PBS) for 60 min before immunostaining using anti-GFP (AbCam ab13970), anti- γ H2AX (Millipore 05-636) as primary- and anti-chicken Alexa 488 (Invitrogen A-11039) and anti-mouse Alexa 594 (Invitrogen A-21203) as secondary antibodies diluted in PTB. Cells were mounted in DAPI containing Vectashield mounting medium (Vector Laboratories). Images were taken using Olympus FV1000 confocal microscope.

### RNAi and stable cell lines

For siRNA knock-down, siRNA duplexes (Ambion) were used. HeLa cells were transfected with the appropriate siRNA duplex (100 pmol/transfection) using Lipfectamine 2000 (Invitrogen) in six-well plates followed by incubation at 37°C for 48 h. The messenger RNA target sequences (sense strand) used for siRNAs were as follows: Spartan 1 (5′-CUA CUU UCC UAG AGU AUC A-3′), RAD18 (5′-GUU CAG ACA UCA UAA GAG A-3′); Spartan 2 (5′-GGA UGU GAG UGG GUC UGA A-3′) and Spartan 3 (5′-CAA GGA UAA GUG UAA CAG U-3′).

For generating USP1-specific shRNA, we cloned the USP1-specific DNA sequence O2793 (5′-GAT CTT CGG CAA TAC TTG CTA TCT TAC AAG AGA TAA GAT AGC AAG TAT TGC CGA TTT TTA-3′) (36) into the BglII-HindIII site of the shRNA-Neo plasmid (37), resulting in pIL2409.

For generating Spartan-specific shRNA expressing stable cell lines, we cloned the Spartan-specific DNA sequence O2689 (5′-AGC TTC TAC TTT CCT AGA GTA TCA TTC AAG AGA TGA TAC TCT AGG AAA GTA GTT TTT TG-3′) in the HindIII site of the shRNA-Neo plasmid, resulting pIL2245. Next, pIL2245 was transfected into HeLa cells followed by selection using G-418 SULPHATE (Gibco, Cat. No.: 11811064) for obtaining stable cell lines.

To generate a FLAG-Spartan-expressing stable cell line, the WT Spartan from plasmid pIL1783 was cloned into a FLAG-Hygro plasmid resulting in plasmid pIL2240. For obtaining a FLAG-Polη expressing stable cell line, the Polη cDNA from plasmid pIL1399 was cloned into plasmid Flag-Hygro resulting in plasmid pIL1967. Next, the FLAG-Spartan-Hygro and FLAG-Polη-plasmids were transfected into HeLa cells followed by selection using hygromicin (Invitrogen A9277) to obtain stable cell lines.

HCT116 RAD18 -/- cell line was described previously (38). To complement the HCT116 RAD18 -/- cells, we used a DsRed-Rad18 plasmid construct.

### Sensitivity assay

HeLa cells were transfected with 100 pmol siRNAs using Lipofectamine 2000 (Invitrogen) in six-well plates. Cell competition-based sensitivity assay was performed as described earlier (39). Briefly, after 24 h, the cells were mixed with stable GFP expressing HeLa cells with 1:1 ratio, which cell mixture was treated in the following day with UV or MMS as indicated. After 7 days of culturing, the ratio of GFP negative and positive cells (surviving cells) was determined by FACS (Guava Easy site System).

### Immunoprecipitation

Cells (2.5 × 10^6^) were lysed in a buffer containing 50 mM Tris–HCl (pH 7.5), 5 mM EDTA, 150 mM NaCl, 0.1% NP-40, 1 mM PMSF and protease inhibitor cocktail (Sigma P8340). To reduce viscosity, the cell lysates were sonicated on ice. FLAG-tagged proteins were immunoprecipitated with immobilized M2 FLAG antibodies (Sigma). The precipitated proteins and the input lysates were analyzed by Western blot using mouse anti-PCNA HRP (Santa cruz cat. numb. sc-56), mouse anti-FLAG HRP (Sigma M2 A8592), rat anti-HA HRP (Roche, cat.numb. 12013819001 clone 3F10), rabbit anti-Tubulin (Santa cruz cat. numb. sc-9104) and goat anti-rabbit HRP (Millipore AP132P) antibodies.

### Sister chromatid exchange analysis

Sister chromatid exchange (SCE) analysis was carried out as described before (40). Briefly, the cells were propagated in dark for two cycles in 30 µM bromodeoxyuridine (BrdU) containing medium followed by culturing in colcemid (Sigma, cat. numb. D1925) for 2 h. Next, cells were collected and treated with 75 mM KCl for 5 min before fixing with methanol for 2 min followed by suspending in methanol-acetic acid (3:1). The cell suspension was dropped onto glass slides and air dried. The chromosomes on the slides were stained with acridine orange (0.1 µg/ml) (Sigma, Cat. No. A8097) before microscopy.

## RESULTS

### Domain structure of Spartan

**Figure 1.**
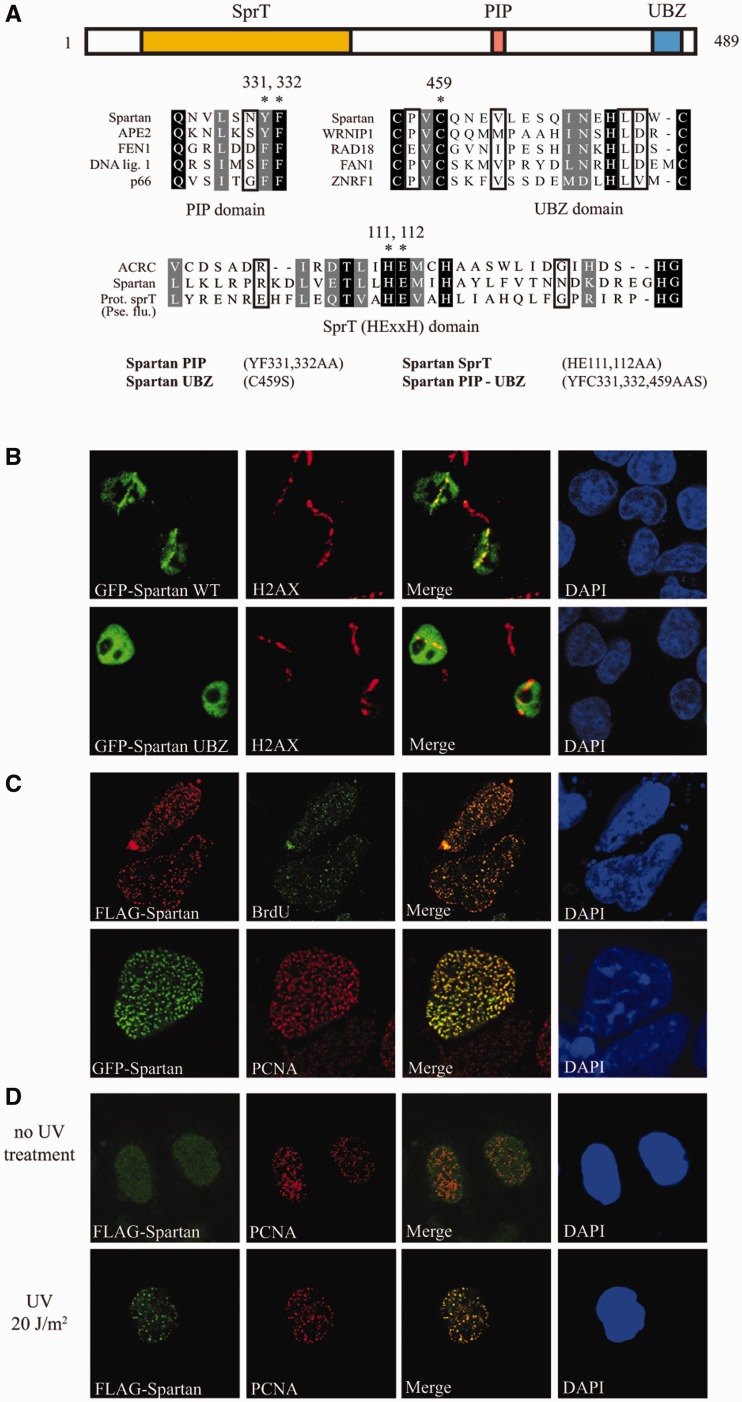
Domain structure and localization of Spartan to stalled replication forks. (**A**) Schematic representation of the domain architecture of human Spartan. Conserved domains are indicated: SprT, putative metalloprotease domain, PIP, PCNA interacting peptide motif; UBZ, ubiquitin-binding zinc-finger domain. In the multiple alignment of the PIP, UBZ and SprT domains of selected proteins, the identical residues are shaded in black, and similar residues are indicated in gray. Asterisks indicate residues mutated in subsequent experiments; the names of the generated point mutant Spartan proteins are indicated. (**B**) Recruitment of Spartan to local DNA damage sites. HCT116 cells transiently expressing GFP-Spartan or the GFP-Spartan UBZ mutant were subjected to laser microirradiation, and 20 min later, immunostaining was performed on them using anti-GFP (green) and anti-γ H2AX (red) antibodies, and the nuclei were stained with DAPI. (**C**) Localization of Spartan to sites of DNA replication. The sites of DNA synthesis of HeLa cells transiently expressing FLAG-Spartan were pulse labeled with BrdU and processed for indirect immunoflourescence with antibodies against FLAG and BrdU (upper panel). Similarly, HeLa cells transfected with GFP-Spartan expressing plasmids were processed for imaging of GFP-Spartan and PCNA (lower panel). (**D**) Localization of Spartan to DNA damage sites. Cells of an isolated HeLa cell line stably expressing a low concentration of FLAG-Spartan were mock treated or irradiated with 20 J/m^2^ UV light. After the treatment, cells were cultivated for 3 h and immunostained with antibodies against FLAG and PCNA.

Spartan/C1orf124 contains a UBZ-type ubiquitin-binding domain (41,42) that belongs to the RAD18 family of zinc fingers (Figure 1A), and we suspected that Spartan is involved in DNA damage response for several reasons. First, all the examined proteins containing its type of UBZ domain such as RAD18, Mgs1 and FAN1 play a role in DDT. Another hint was the presence of a highly conserved PCNA interacting protein motif (PIP) in Spartan beside a SprT domain found in the bacterial SprT, a metallopeptidase-like protein (Figure 1A). Moreover, we found in the Tumorscape Database (43), which reveals chromosomal regions across different cancer types harboring undiscovered genes whose copy number variations may be cancer-causing, that Spartan is significantly deleted across a data set of 3131 different tumors analyzed, suggesting a possible role of Spartan in cancer suppression. We therefore decided to test whether Spartan is involved in DNA damage response.

### DNA damage-induced recruitment of Spartan to sites of DNA replication

To examine whether Spartan could be recruited to DNA damage sites, we subjected GFP-tagged Spartan cells to laser microirradiation, which results in localized DNA damage tracks (35) (Figure 1B). Twenty minutes after irradiation, GFP-Spartan localized to sites of DNA damage along with γ-H2AX, a marker of DNA damage sites. We also tested the UBZ-domain mutant of Spartan and found that it completely failed to localize to microirradiation tracks (Figure 1B). These observations suggested that Spartan foci formation might depend on association with an ubiquitylated protein at DNA damage sites.

We next asked whether Spartan localizes to the sites of ongoing DNA synthesis. During DNA replication, PCNA accumulates in focal structures indicative of sites of DNA synthesis, which can also be visualized by immunostaining for BrdU after BrdU incorporation into newly replicated DNA facilitated by pulse labeling. Transient, high level expression of FLAG-Spartan or GFP-Spartan resulted in nearly complete colocalization of Spartan with PCNA and BrdU even in non-damaged cells (Figure 1C). However, when we generated cell lines stably expressing low levels of FLAG-Spartan, we found that Spartan was distributed uniformly in the nucleus, and its redistribution to PCNA-containing foci required treatment with agents that can induce replication stalling such as UV radiation or MMS (Figure 1D and Supplementary Figure S1). We conclude that DNA damage induces the recruitment of Spartan to the sites of stalled replication forks.

### Spartan recruitment depends on its PCNA and UBZ domains and the RAD18 PCNA ubiquitin ligase

**Figure 2.**
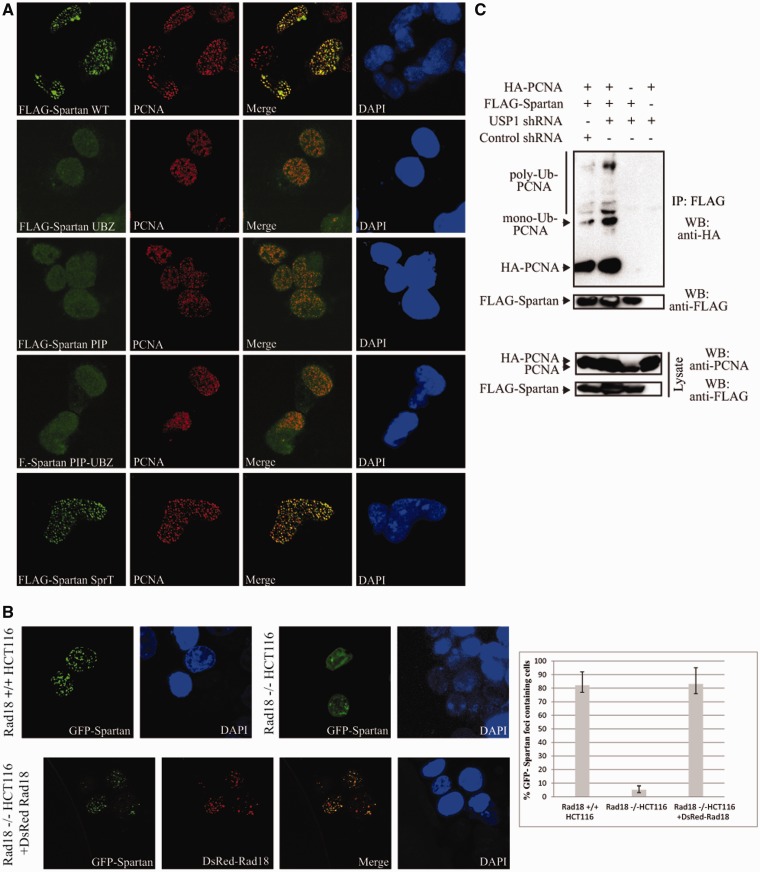
Requirement of the UBZ and PIP domains of Spartan and Rad18 for Spartan localization to replication forks. (**A**) The UBZ and PIP domains of Spartan are essential for foci formation and colocalization of Spartan with PCNA. HeLa cells transiently transfected with plasmids expressing WT, UBZ, PIP, PIP-UBZ or SprT domain mutant FLAG-Spartan proteins were processed for indirect immunofluorescence with antibodies against FLAG and PCNA. (**B**) The localization of Spartan depends on Rad18. Knock out HCT116 RAD18-/- and RAD18+/+ cells were transfected with plasmids expressing GFP-Spartan of which localization was compared in the two cell lines (upper panel). Localization of GFP-Spartan in RAD18-/- cells and DsRed-Rad18 expressing RAD18-/- cells (lower panel) was also compared. The percentages of GFP-Spartan expressing cells that display more than five Spartan foci were determined from three independent experiments and standard deviation was also calculated (right panel). (**C**) Spartan associates with mono- and polyubiquitin PCNA *in vivo*. HEK 293 cells were transfected with various combinations of control or USP1 shRNAs, HA-PCNA and FLAG-Spartan. Cell extracts were subjected to immunoprecipitation with anti-FLAG antibody, and the coimmunoprecipitated unmodified-, mono- and polyubiquitinated PCNAs were detected by western blotting using anti-HA antibody.

Next we tested the contribution of the SprT, PIP and UBZ domains of Spartan for its recruitment to replication foci. Although the SprT domain mutant Spartan retained the localization properties of WT Spartan, the Spartan proteins of the UBZ and PIP domain mutants completely failed to localize to PCNA-marked replication forks (Figure 2A), suggesting that PCNA binding and ubiquitin binding by Spartan might be important for its recruitment.

To test whether ubiquitylated PCNA is the recruiting factor for Spartan, we asked if Rad18, an ubiquitin ligase responsible for damage-induced PCNA monoubiquitylation, was required for foci formation of Spartan. Thus, we compared the localization of GFP-Spartan in RAD18 proficient and deficient cell lines (Figure 2B). Supporting the importance of PCNA ubiquitylation, in RAD18-/- knock out cells (38), Spartan completely failed to form foci when compared with its proficient foci formation in RAD18+/+ cells; however, expression of DsRed-Rad18 in RAD18-/- cells restored Spartan foci formation and, notably, revealed complete colocalization of Spartan with Rad18 (Figure 2B). Moreover, transient depletion of RAD18 by siRNA in another human cell line also led to impaired Spartan foci formation (Supplementary Figure S2A and S2B). In line with these finding, Spartan can be coimmunoprecipitated with Rad18 (Supplementary Figure S2C). To provide evidence for the *in vivo* interaction of Spartan with ubiquitylated PCNA, we found that Spartan can also be coimmunoprecipitated with ubiquitylated PCNA (Figure 2C). Furthermore, when we depleted USP1, which has been shown to increase endogenous ubiquitin-conjugated PCNA species (24), an increased level of ubiquitylated PCNA could be pulled down by Spartan (Figure 2C). These results together suggested that Spartan is recruited to stalled forks by ubiquitin-conjugated PCNA.

### Purified Spartan directly and preferentially binds to ubiquitin-conjugated PCNA

**Figure 3.**
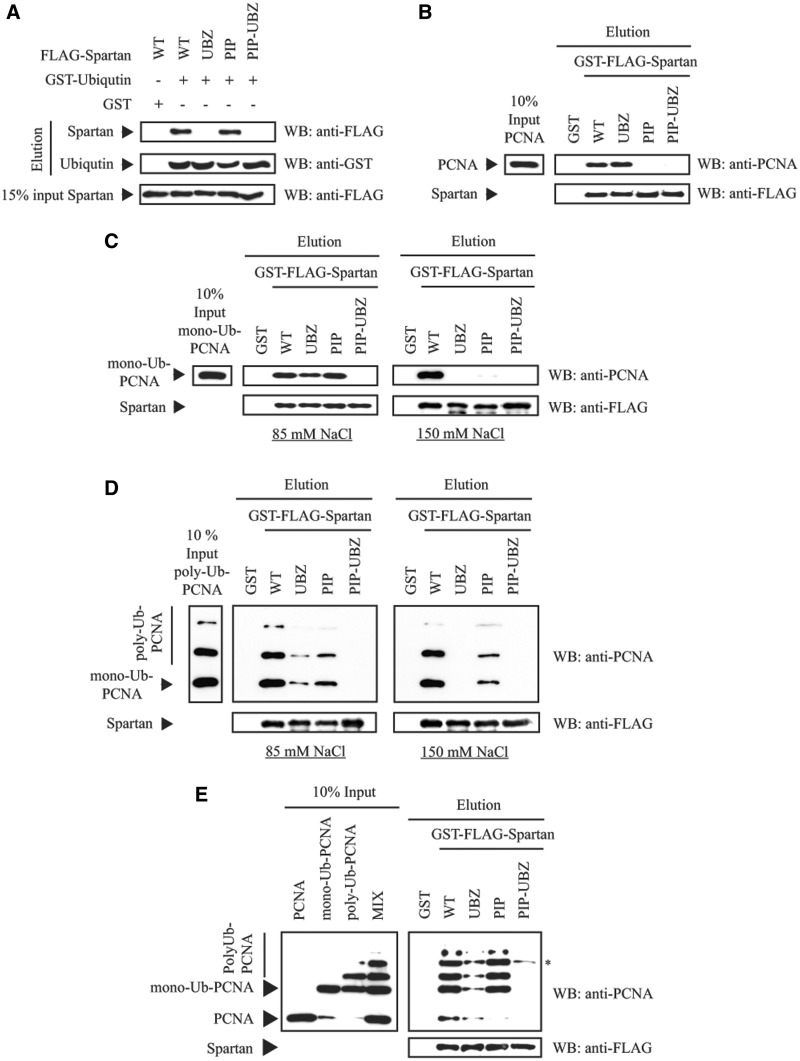
Purified Spartan preferentially binds to ubiquitin-conjugated PCNA. (**A**) The UBZ domain of Spartan mediates ubiquitin binding. GST-ubiquitin-bound glutathione-sepharose beads were incubated with purified WT, UBZ, PIP or PIP-UBZ mutant FLAG-Spartan in an 85 mM NaCl containing buffer. After elution, samples were analyzed for direct physical interaction of Spartan and ubiquitin with anti-FLAG and anti-GST antibodies after western blotting. (**B**) The PIP domain of Spartan mediates PCNA binding. Purified WT, UBZ, PIP or PIP-UBZ mutant GST-FLAG-Spartan samples were immobilized on glutathione-sepharose and incubated with PCNA in an 85 mM NaCl containing buffer. Eluted samples were analyzed for complexes of Spartan and PCNA by western blotting with anti-PCNA and anti-FLAG antibodies. (**C**) Requirement of the PIP- and UBZ domain of Spartan for monoubiquitin-PCNA binding. As described in (B) but bead-bound Spartan was incubated with purified monoubiquitin-PCNA instead of unmodified PCNA. Both the incubation and the washing steps were carried out at 85 mM (left panel) and at 150 mM NaCl concentrations (right panels) as indicated. (**D**) Requirement of the PIP- and UBZ domain of Spartan for polyubiquitin-PCNA binding. As described in (C) but bead-bound Spartan was incubated with polyubiquitin-PCNA. (**E**) Preferential association of Spartan with ubiquitin-conjugated PCNA. As described in (B) but bead-bound WT and mutant Spartan proteins were incubated with a mixture of equal amount of unmodified PCNA, monoubiquitin-PCNA and polyubiquitin-PCNA.

To explore whether Spartan can directly associate with ubiquitin-conjugated PCNA, we used purified WT, UBZ-, PIP- and PIP-UBZ double mutant Spartan proteins along with purified ubiquitin, PCNA and *in vitro* mono- and polyubiquitylated PCNA in pull-down assays using less stringent salt concentration (85 mM NaCl) to reveal also weaker interactions and higher, close to physiological salt concentration (150 mM NaCl). First, Spartan was incubated with GST-tagged ubiquitin bound to glutathione beads. As shown in Figure 3A, WT Spartan strongly associated with ubiquitin, whereas the UBZ mutant Spartan failed to bind ubiquitin; however, the PIP mutant Spartan was proficient in ubiquitin binding. Next, PCNA was incubated with GST-FLAG-tagged Spartan bound to glutathione beads, which revealed that Spartan can directly bind to PCNA and that mutation in the PIP-box but not in the UBZ domain of Spartan impaired their association (Figure 3B). In a similar assay at 85 mM NaCl concentration, we found that Spartan strongly associated with monoubiquitin-PCNA, and only the PIP-UBZ double mutant Spartan but not the single UBZ or PIP mutant failed completely in this interaction (Figure 3C). However, at 150 mM NaCl, neither the PIP nor the UBZ mutant Spartan could interact with monoubiquitin-PCNA, which is consistent with our localization studies indicating that both the PIP box and the UBZ domain in Spartan are essential for Spartan colocalization with PCNA (Figure 3C, right panel). Essentially the same result was obtained using polyubiquitin PCNA instead of monoubiquitin-PCNA (Figure 3D). Finally, to compare the binding affinity of Spartan to unmodified and ubiquitin-conjugated PCNA, we mixed equal amounts of unmodified PCNA, monoubiquitin-PCNA and polyubiquitin-PCNA and incubated this mixture with GST-FLAG-Spartan bound to gluthatione beads (Figure 3E). Notably, Spartan preferably pulled down mono- and polyubiquitylated PCNA over unmodified PCNA, and the PIP-UBZ double mutant Spartan completely failed to pull down ubiquitin-conjugated PCNA and unmodified PCNA (Figure 3E). These observations suggest that the UBZ and PIP domains of Spartan cooperatively ensure a strong preferable binding to ubiquitin-modified PCNA.

### Spartan depletion sensitizes human cells to DNA damaging agents

**Figure 4.**
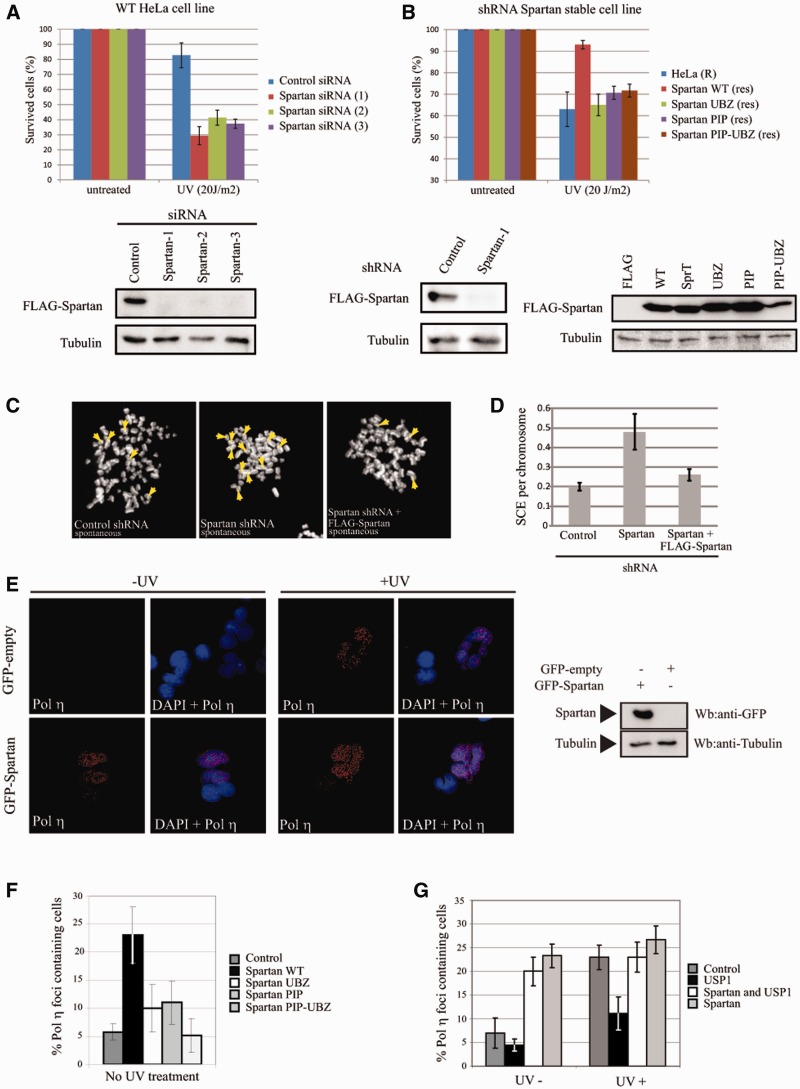
Role of Spartan in DNA damage tolerance. (**A**) Spartan depletion sensitizes human cells to UV-irradiation. HeLa cells treated with control or three different siRNAs were assayed for survival after UV treatment by cell competition assay using a reference GFP+ HeLa cell line. The results of three independent experiments for each sample with standard deviation are graphed. Efficiency of siRNA depletion of Spartan was tested with anti-FLAG antibody after 48 h of cotransfecting the FLAG-Spartan plasmid and three different types of Spartan siRNAs. (**B**) UBZ and PIP domains of Spartan are all required for damage resistance. Complementation of the UV sensitivity of stable Spartan depleted HeLa cells by shRNA resistant form of WT, UBZ, PIP and PIP-UBZ domain mutant FLAG-Spartan (upper panel). The efficiency of stable shRNA Spartan depletion was assayed as in (A), and shRNA-resistant WT or point mutant FLAG-Spartan expressions were confirmed with anti-FLAG antibody (lower panels). (**C**) The silencing of Spartan causes increased SCE. The control and Spartan depleted stable HeLa cells were compared in spontaneous SCE analysis. (**D**) Quantification of the SCE. SCE was compared in the following three different stable HeLa cells: 1, expressing control shRNA; 2, Spartan depleted: 3, Spartan depleted but expressing shRNA-resistant FLAG-Spartan. SCE per chromosome values were determined by counting one hundred cells per sample. The results of three independent experiments with standard deviations are graphed. (**E**) Spartan facilitates Polη foci formation. FLAG-Polη expressing stable HeLa cells were transfected with GFP or GFP-Spartan expressing plasmids, and after 24 h cultivation, mock or UV (20 J/m^2^) treated. After extracting cells with NP40, a non-ionic detergent, localization of Polη was visualized by immunostaining using anti-FLAG antibody. The expression level of GFP-Spartan was confirmed by anti-GFP antibody after western blotting of cell extracts (left panel). (**F**) Stimulation of Polη foci formation depends on the UBZ and PIP domains of Spartan. FLAG-Polη expressing stable HeLa cells was transfected with WT, UBZ, PIP and PIP-UBZ GFP-Spartan expressing constructs followed by quantitation for Polη foci forming cell when compared with the green transfected cells. The cells with more than five GFP-Spartan foci were counted as foci positive. (**G**) Competition of Spartan and USP1 on Polη foci formation. USP1, or Spartan together with USP1, or Spartan was expressed in HeLa cells stably expressing FLAG-Polη, and the percentage of Polη foci forming cells was quantitated. The results of three independent experiments with standard deviations are graphed.

Given that Spartan binds to ubiquitin-conjugated PCNA and localizes to Rad18-marked stalled replication forks, we asked whether Spartan deficiency, similarly to deficiency in RAD18 or PCNA ubiquitylation (44), sensitizes cells to DNA damage, and if so, whether the UBZ and PIP domains of Spartan are crucial for conferring damage resistance. To this end, we knocked down Spartan with three different siRNAs targeting different regions of Spartan mRNA and subjected these cells to treatment with DNA-damaging agents. When compared with the control siRNA-treated cells, the Spartan-depleted cells were hypersensitive to UV-irradiation and the methylating agent MMS (Figure 4A and Supplementary Figure S3). We also generated a stable Spartan knock-down cell line, whose sensitivity to UV could be complemented with a shRNA-resistant WT Spartan, which rules out the off-targeting of shRNA toward an unrelated protein (Figure 4B). In contrast, shRNA-resistant UBZ- or PIP-domain mutant Spartan could not complement the impaired UV resistance of these stable Spartan-depleted cells, indicating that the ubiquitin and PCNA binding of Spartan is indispensable for Spartan function in providing protection against genotoxic agents (Figure 4B).

### Depletion of Spartan results in increased sister chromatin exchange

RAD18-dependent PCNA ubiquitylation has been shown to channel the rescue of stalled forks from recombination to damage bypass by TLS or template switching (23). Taking the interaction of Spartan with ubiquitin-conjugated PCNA into account, we asked whether Spartan could also have a similar channeling function. Recombination between sister chromatids can result either in crossover events, which can be visualized on metaphase chromosomes as SCEs, or in non-crossover events. As impairment in RAD18 can increase even spontaneous SCEs formed presumably at endogenous lesions (45), we tested the effect of Spartan depletion on spontaneous SCE. To this end, control and stable Spartan-depleted cells were tested by labeling with BrdU for one round of DNA replication followed by visualization of the exchanges between sister chromatids on isolated mitotic chromosomes by orange acridine staining, which distinguishes DNA strands with and without incorporated BrdU. As shown in Figure 4C and D, stable Spartan depletion led to a 2.5-fold increase in the number of SCEs. To further verify, we expressed shRNA-resistant Spartan in stable Spartan-depleted cells and found complementation of SCEs formation (Figure 4D). We conclude that Spartan prevents the formation of crossover events between sister chromatids.

### Spartan facilitates the recruitment of Polη to replication foci

To provide evidence that Spartan can indeed channel fork rescue to damage bypass, we examined whether the access of a chosen TLS polymerase to replication forks can be affected by an increased Spartan expression level. Polη has been shown to be distributed uniformly throughout the nucleus, from which it can be extracted by a mild washing with a non-ionic detergent; however, upon UV irradiation, Polη colocalizes with monoubiquitylated PCNA in replication foci and becomes resistant to non-ionic detergent extraction. First, we examined whether transient expression of GFP-Spartan in FLAG-Polη-expressing stable HeLa cells affects localization of Polη to replication foci. Notably, we found that transient expression of Spartan highly increased Polη foci formation and also eliminated the requirement of UV-irradiation (Figure 4E and F and Supplementary Figure S3B). This effect of Spartan was impaired by mutation in UBZ- or PIP domains of Spartan, emphasizing the importance of its ubiquitin-PCNA binding and ruling out that this observation is indirect, for example, due to inducing DNA damage by transient overexpression of Spartan (Figure 4F and Supplementary Figure S3C).

To provide additional confirmation, we aimed to test the effect of decreasing the level of ubiquitin-conjugated PCNA, which can be carried out by expressing the PCNA-deubiquitylating enzyme USP1 as described. As expected, transient expression of USP1 resulted in decreased Polη foci formation, but strikingly, this was suppressed by Spartan expression, because expression of USP1 together with Spartan resulted in an increased number of Polη foci that was comparable to the foci number observed during expression of Spartan alone (Figure 4G). Thus, Spartan and USP1 have opposing functions in providing access of Polη to replication forks. To explain this finding, one possibility was that Spartan stimulates PCNA ubiquitylation; however, we considered this unlikely because Spartan recruitment required preexisting ubiquitin-conjugated PCNA. The other more likely possibility is that Spartan and USP1 can compete for the access to ubiquitylated-PCNA and that Spartan is an antagonist of USP1-dependent PCNA deubiquitylation.

### Spartan inhibits the USP1-dependent deubiquitylation of ubiquitin-conjugated PCNA

**Figure 5.**
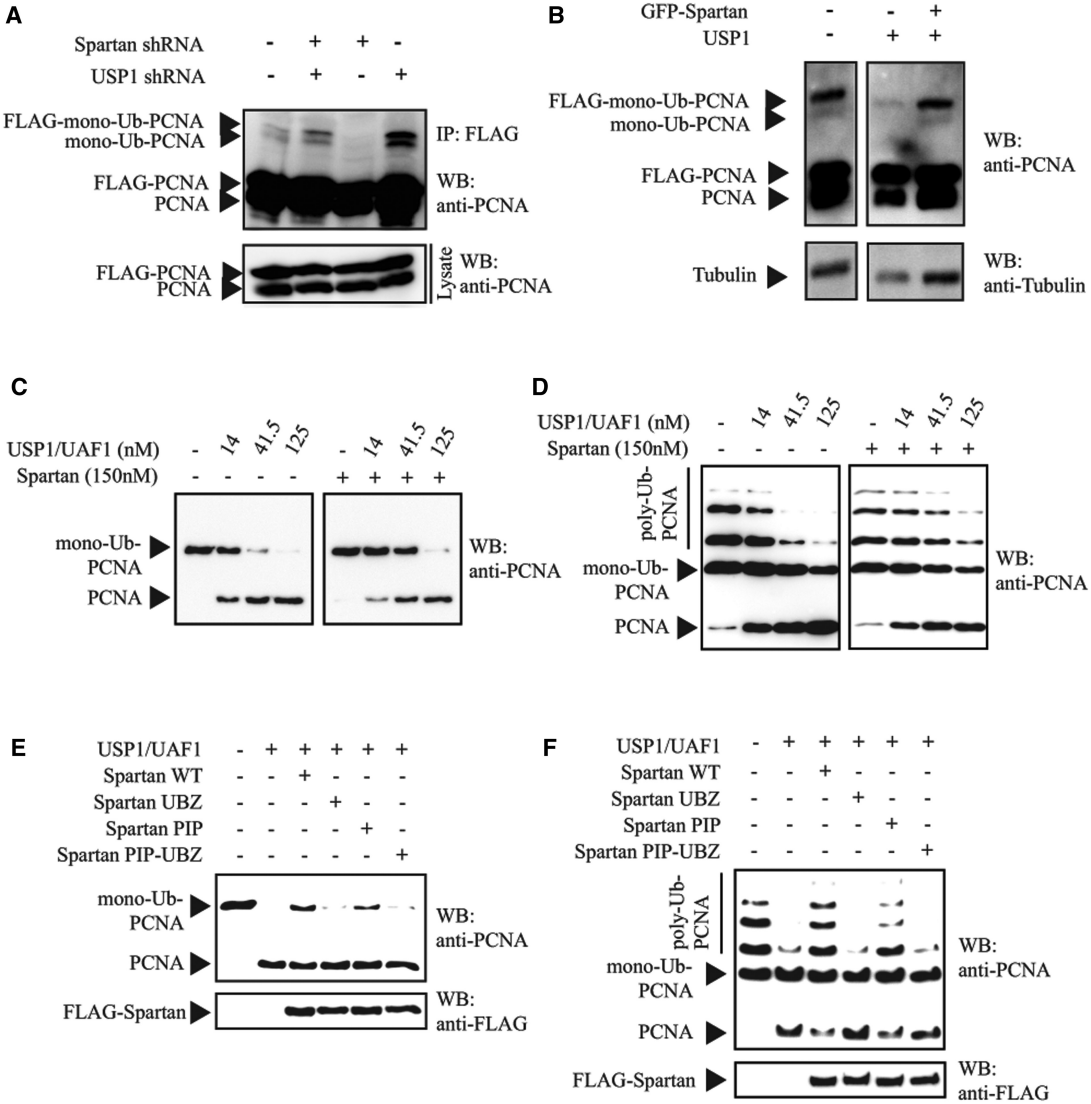
Inhibition of the USP1-dependent deubiquitylation of ubiquitylated PCNA by Spartan. (**A**) USP1 knockdown reverses the reduction of PCNA monoubiquitination caused by Spartan knockdown. Spartan, USP1 and they together were knockdown along with transient expression of FLAG-PCNA in HEK 293 cells. After 24 h of transfection, cells were irradiated with 20 J/m^2 ^UV, and in 3 h, cell extracts were subjected to immunoprecipitation with anti-FLAG antibody. Monoubiquitylation of endogenous and FLAG-PCNA was detected by western blotting using anti-PCNA antibody. (**B**) Spartan overexpression reverses the reduction of PCNA monoubiquitylation caused by USP1 overexpression. HEK 293 cells were transfected with FLAG-PCNA and USP1 expression constructs together with mock or Spartan expression constructs. Cell extracts were prepared and analyzed as described in (A). (**C**) Spartan inhibits USP1-UAF1-dependent *in vitro* deubiquitylation of monoubiquitin-PCNA. Increasing amounts of purified USP1-UAF1 were incubated with purified monoubiquitin-PCNA in the absence (Lanes 1–4) or presence (5–8) of Spartan at 37°C for 45 min. Deubiquitylation of PCNA was analyzed on 10% denaturing polyacrilamyde gels followed by western blotting and visualization with anti-PCNA antibody. (**D**) Spartan inhibits USP1-UAF1-dependent *in vitro* deubiquitylation of polyubiquitin-PCNA. Analysis was carried out as in (A) but using purified polyubiquitin-PCNA substrate instead of monoubiquitin-PCNA. (**E**) The UBZ and PIP domains of Spartan are essential for inhibition of USP1-UAF1-dependent deubiquitylation of monoubiquitin-PCNA. Monoubiquitin-PCNA was incubated with USP1-UAF1 (50 nM) in the absence or presence of WT, UBZ, PIP or PIP-UBZ mutant Spartan proteins (150 nM). The reaction products were analyzed for deubiquitylation of monoubiquitin-PCNA by western-blotting using anti-PCNA antibody. (**F**) The UBZ and PIP domains of Spartan are essential for inhibition of USP1-UAF1-dependent deubiquitylation of polyubiquitin-PCNA. Analysis was carried out as in (C) but using purified polyubiquitin-PCNA substrate instead of monoubiquitin-PCNA.

To test whether Spartan inhibits USP1-dependent PCNA deubiquitylation, first we examined the consequences of Spartan and USP1 knockdown and of their overexpression on the level of monoubiquitin-PCNA (Figure 5A and B). USP1 knockdown, as expected, led to a higher monoubiquitin-PCNA level, whereas Spartan knockdown resulted in a much lower cellular concentration of monoubiquitin-PCNA, which indicates that Spartan and USP1 have opposite influence on the level of monoubiquitin-PCNA. Importantly, however, combining Spartan knockdown with USP1 knockdown reversed the reduction of PCNA monoubiquitination (Figure 5A). Furthermore, although USP1 overexpression decreased the amount of monoubiquitin-PCNA, its effect was suppressed by simultaneous overexpression of Spartan (Figure 5B). These results are consistent with that Spartan functions as an antagonist of PCNA deubiquitylation by USP1.

To examine directly whether Spartan can protect ubiquitin-PCNA against deubiquitylation by USP1, we carried out a series of deubiquitylation assays of mono- and polyubiquitin-PCNA by USP1-UAF1 in the presence or absence of Spartan *in vitro* using highly purified proteins (Figure 5C–F). As shown in Figure 5C and D, *in vitro* USP1-UAF1 could indeed deubiquitylate mono- and polyubiquitin PCNA in a concentration-dependent manner, resulting in unconjugated PCNA. Importantly, adding Spartan to the PCNA deubiquitylation reaction strongly inhibited PCNA deubiquitylation by USP1-UAF1 (Figure 5C and D). The PIP mutant Spartan prevented PCNA deubiquitylation less efficiently when compared with the WT Spartan. Moreover, the UBZ mutant Spartan completely failed to protect mono- and polyubiquitin PCNA against USP1-catalyzed deubiquitylation (Figure 5E and F). These observations suggest that the PCNA-binding and the ubiquitin-binding properties of Spartan contribute to the protection against PCNA deubiquitylation.

## DISCUSSION

In this work, we characterized Spartan, a previously unknown protein, and unraveled its role in the RAD18-dependent pathway of replication of damaged DNA. Upon DNA damage, Spartan localizes to sites of replication stress in a Rad18-dependent manner and also interacts with Rad18. Several lines of evidence suggest that the recruitment of Spartan to replication forks is mediated by the Rad18-dependent ubiquitylation of PCNA. First, purified Spartan preferentially interacts with ubiquitin conjugated PCNA, which is facilitated by the PIP- and UBZ domains of Spartan, and Spartan also forms complex with ubiquitin-modified PCNA *in vivo*. Next, colocalization of Spartan with PCNA requires the UBZ and the PIP domains of Spartan. Finally, binding of purified Spartan by its PIP- and UBZ domains to ubiquitin-conjugated PCNA provides protection against PCNA deubiquitylation by USP1.

In addition to monoubiquitin-PCNA, Spartan can also preferentially bind to polyubiquitin-PCNA and protect against USP1-dependent removal of its polyubiquitin chain. This finding provides a strong base for further studies aiming to explore polyubiquitin-PCNA and its deubiquitylation-dependent regulation of template switch-dependent replication of damage DNA.

**Figure 6.**
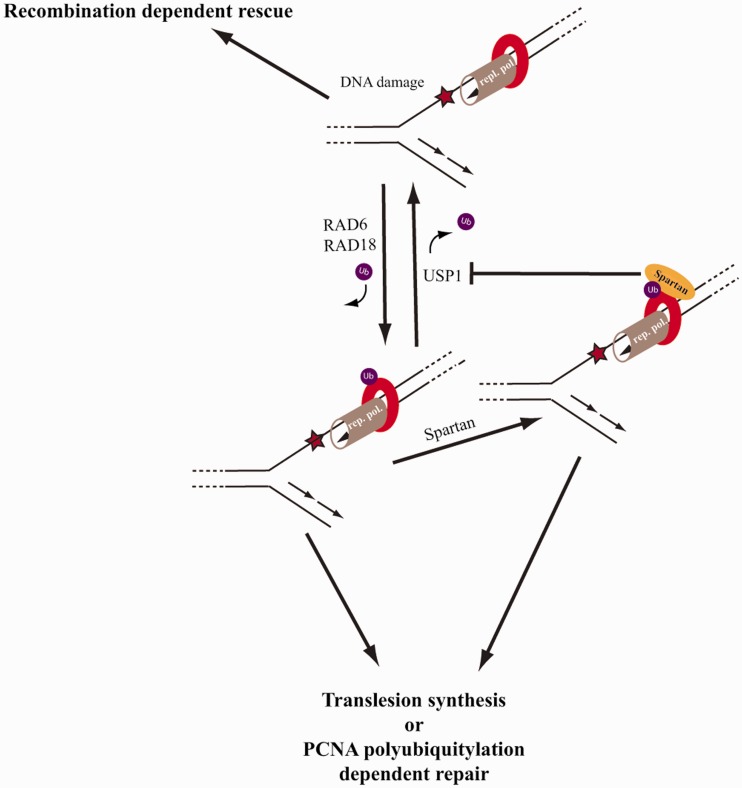
Model for the role of Spartan in DNA damage tolerance. We suggest that at the stalled replication fork, the Rad6-Rad18-dependent PCNA ubiquitylation and the USP1-dependent PCNA deubiquitylation are dynamic processes, of which balance determines the life-time of ubiquitin-PCNA and the choice of fork rescue mechanism. In the absence of ubiquitin-PCNA, replication fork can be rescued by recombination-dependent mechanisms, which, however, have a potential for DNA rearrangements. In the presence of ubiquitin-conjugated-PCNA, damage bypass or template switching can provide replication through the lesion without the formation of a DSB intermediate. Spartan can provide one regulatory level by binding to ubiquitin-modified PCNA, which protects against PCNA deubiquitylation by USP1. Thus, Spartan can channel the reviving of stalled replication from a recombination-dependent pathway that does not require PCNA ubiquitylation to PCNA ubiquitylation-dependent translesion synthesis or PCNA polyubiquitylation-dependent template switching pathways.

During the course of the study, Spartan/C1orf124 was also found to be recruited to stalled replication forks by ubiquitylated PCNA and shown to enhance TLS, with which our study fully agrees (33). However, it was suggested that Spartan might stimulate PCNA ubiquitylation by enhancing Rad18 ubiquitin ligase function, which we consider unlikely for several reasons. First, we found that the reduction of monoubiquitin-PCNA level caused by Spartan knockdown could be reversed by simultaneous USP1 knockdown. Also, Spartan overexpression can reverse the USP1 overexpression-dependent reduction of monoubiquitin-PCNA level. Furthermore, in our reconstituted system using purified proteins, Spartan could directly inhibit USP1-dependent PCNA deubiquitylation. Finally, we reason that the Rad18-dependent PCNA ubiquitylation should precede the ubiquitin-PCNA-dependent Spartan recruitment. Instead, our observations are consistent with a model proposing that after PCNA ubiquitylation induced by replication stress, Spartan is targeted to ubiquitin-modified PCNA, where it could provide protection against PCNA deubiquitylation (Figure 6). This possibility suggests that Spartan might channel the reviving of stalled replication from recombination-dependent mechanisms that do not require PCNA ubiquitylation to a PCNA ubiquitylation-dependent damage bypass (17,23,46). In addition to showing *in vitro* using purified proteins that Spartan inhibits PCNA deubiquitylation by USP1 a strong support for this model is also provided by our *in vivo* observation that Spartan depletion increases the number of SCEs, whereas overexpression of Spartan stimulates the access of Polη to the replication fork.

Recently, PCNA deubiquitylation by the USP1-UAF1 complex in conjunction with ELG1, which directs the USP1-UAF1 to ubiquitin-PCNA, has emerged as an important regulatory mechanism of damage bypass (24,26). In facilitating PCNA deubiquitylation, ELG1 and USP1-UAF1 have been suggested to initiate the switch from the low-fidelity polymerase to the high-fidelity replicative polymerases (26). To further support this model, loss of USP1 was shown to lead to aberrant recruitment of TLS polymerase κ to replication forks resulting in genomic instability (28). Indeed, because of the error-prone nature of DNA damage bypass and the low fidelity of TLS polymerases, PCNA ubiquitylation and deubiquitylation need to be tightly regulated. One level of regulation is provided by UV irradiation-dependent inactivation of USP1 through an autocleavage event (24). However, it is unclear what regulates USP1 activity when the protein is not actively degraded following other types of DNA damage (25). Our study suggests a new regulatory mechanism by revealing that Spartan can antagonize USP1-dependent PCNA deubiquitylation. Because the rescue of stalled replication forks by uncontrolled recombination could lead to gross chromosomal rearrangements, we propose that by channeling fork rescue to damage bypass Spartan functions as a guardian during replication of damaged DNA. In fact, Spartan gene is significantly deleted in different tumors, implicating that Spartan might be paramount for cancer prevention in human cells (43).

Undoubtedly, gaining more insight into the Spartan-dependent mechanisms will have important implications in understanding how deregulation of the DDT pathways can lead to chromosomal instability and cancer.

## SUPPLEMENTARY DATA

Supplementary Data are available at NAR Online: Supplementary Figures 1–3.

## FUNDING

The Hungarian Science Foundation [OTKA 101225, GOP-1.1.1-11-2011-0026 and GOP-1.1.1-11-2012-0030]; IPA Cross-border Co-operation Programme [HUSRB/1002/214/126]; US National Institutes of Health [R01GM097468 to Z.Z.]. Funding for open access charge: Hungarian Science Foundation [OTKA 101225].

*Conflict of interest statement*. None declared.

## Supplementary Material

Supplementary Data
