# Supplementary material for: Characterization of human Spartan/C1orf124, an ubiquitin-PCNA interacting regulator of DNA damage tolerance
Source: Nucleic Acids Res. 2012 Sep 16;40(21):10795–808. doi: 10.1093/nar/gks850 (PMC3510514; doi:10.1093/nar/gks850)
Supplement: Supplementary Data [file supp_40_21_10795__index.html]

Characterization of human Spartan/C1orf124, an ubiquitin-PCNA interacting regulator of DNA damage tolerance — Characterization of human Spartan/C1orf124, an ubiquitin-PCNA interacting regulator of DNA damage tolerance — Supplementary Data 

# Characterization of human Spartan/C1orf124, an ubiquitin-PCNA interacting regulator of DNA damage tolerance

## Supplementary Data

files

**Files in this Data Supplement:**

- Supplementary Data - pdf file
