# Supplementary material for: Characterization of human Spartan/C1orf124, an ubiquitin-PCNA interacting regulator of DNA damage tolerance
Source: Nucleic Acids Res. 2012 Sep 16;40(21):10795–808. doi: 10.1093/nar/gks850 (PMC3510514; doi:10.1093/nar/gks850)
Supplement: Supplementary Data [file supp_gks850_nar-01581-d-2012-File008.pdf]

Supplementary Data

Characterization of human Spartan/C1orf124, a ubiquitin-PCNA interacting regulator of DNA damage tolerance

Szilvia Juhasz[1], David Balogh[1], Ildiko Hajdu[1,2], Peter Burkovics[1], Mark A. Villamil[3], Zhihao Zhuang[3] and Lajos Haracska[1]*

[1]Institute of Genetics, Biological Research Centre, Hungarian Academy of Sciences, Szeged, Hungary;
[2]Department of Genetics, Howard Hughes Medical Institute, Division of Genetics, Brigham and Women's Hospital, Harvard University Medical School, MA, Boston, USA
[3]Department of Chemistry and Biochemistry, University of Delaware, Newark, Delaware, USA;

* To whom correspondence should be addressed:Tel: +36-62-599666; Fax: +36-62-599666; Email:haracska@brc.hu
Present Address: Lajos Haracska, Biological Research Centre, H-6726 Szeged, Temesvari krt. 62, Hungary

Supplementary Figures

Supplementary Figure S1. MMS induced replication foci formation of Spartan.

FLAG-Spartan stable HeLa cells were treated with 0,02% MMS followed by 3-hour incubation before visualization using anti-FLAG- and anti-PCNA antibodies.

Supplementary Figure S2. Replication foci formation of Spartan depends on RAD18.

(**A**) siRNA knockdown of RAD18 impairs Spartan foci formation. HeLa cells were transfected with a mixture containing GFP-Spartan and either control or RAD18 specific siRNAs and after 24 hr processed for imaging of GFP-Spartan and PCNA.

(**B**) Quantification of the GFP-Spartan focus formation ability in RAD18 siRNA silenced HeLa cells. Graphical representation of the percentage of GFP-Spartan expressing cells that display at least five Spartan foci were calculated from three independent experiments and standard deviation is shown. Efficiency of siRNA depletion of RAD18 was tested with anti-FLAG antibody after 48 hours of cotransfecting the FLAG-RAD18 plasmid and control or Spartan-specific siRNAs (left).

(C) Spartan associates with Rad18 *in vivo*. HEK 293 cells were transfected with the indicated combinations of control, HA-Rad18, and FLAG-Spartan expression plasmids. Cell extracts were subjected to immunoprecipitation with anti-FLAG antibody and the coimmunoprecipitated Rad18 was detected by western blotting using anti-HA antibody.

Supplementary Figure 3.

(A) Spartan depletion sensitizes human cells to MMS. HeLa cells treated with control or three different siRNAs were assayed for survival after MMS treatment by cell competition assay using a reference GFP+ HeLa cell line.

(B) UV-independent foci formation of Polη in the presence of expressed Spartan. HeLa cells were transfected with control or GFP-Spartan expressing constructs followed by mock or UV-treatment before quantitation for Polη foci forming cell as compared to the green transfected cells. The cells with more than five GFP-Spartan foci were counted as foci positive.

(C) siRNA resistant WT or point mutant HA-Spartan expressions were confirmed with anti-HA antibody (control experiment for Figure 4F).

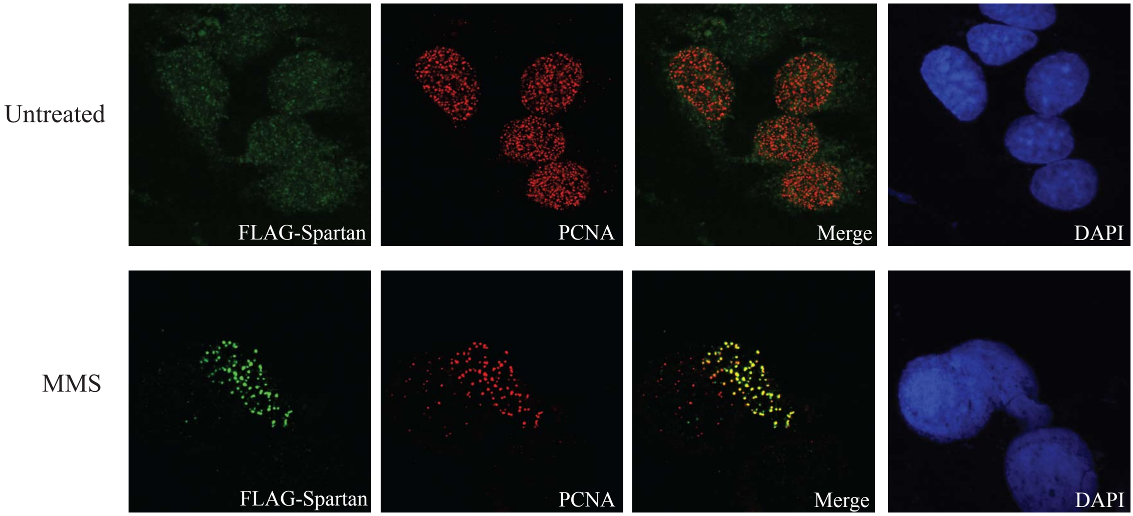

Supplementary Figure 1

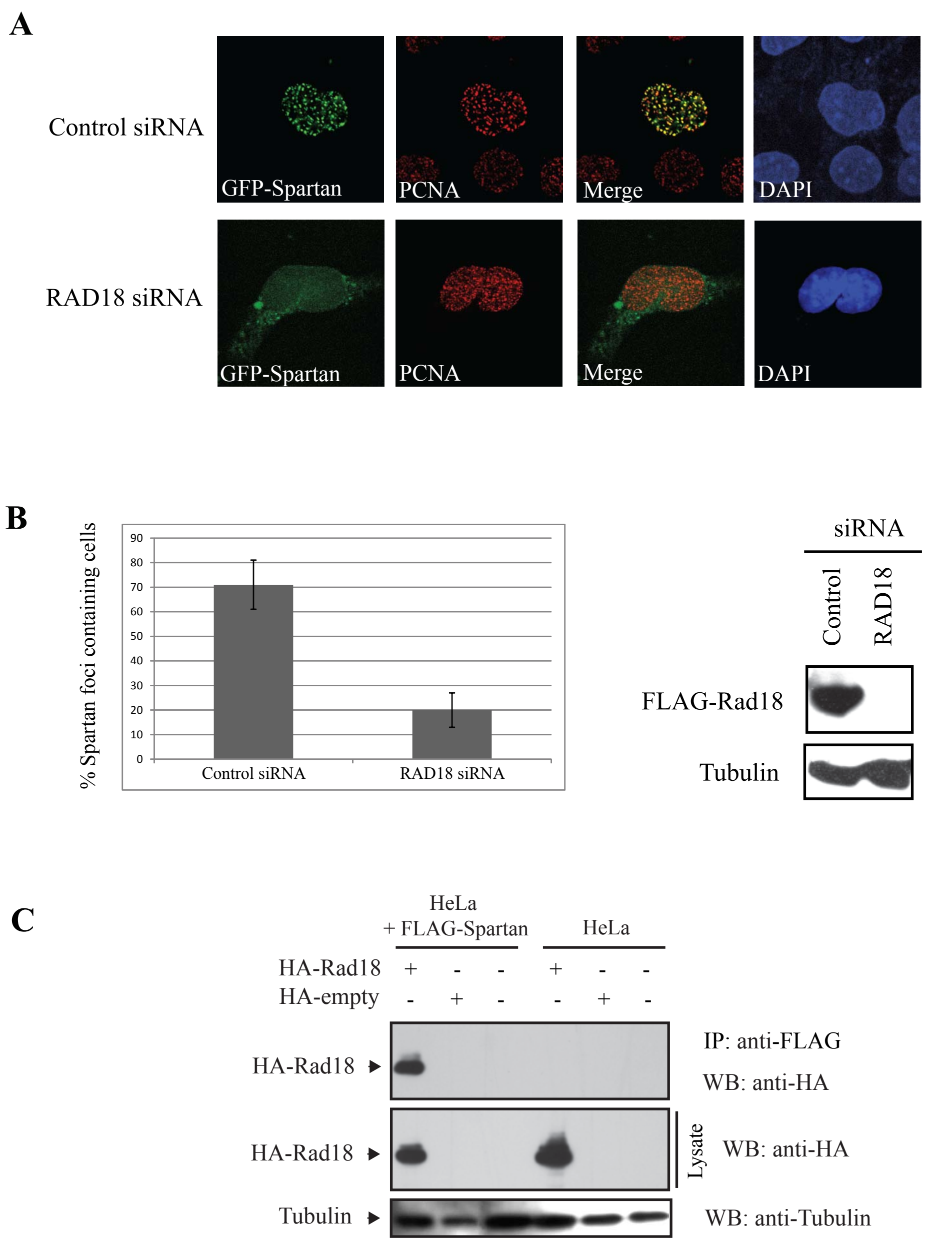

Supplementary Figure 2

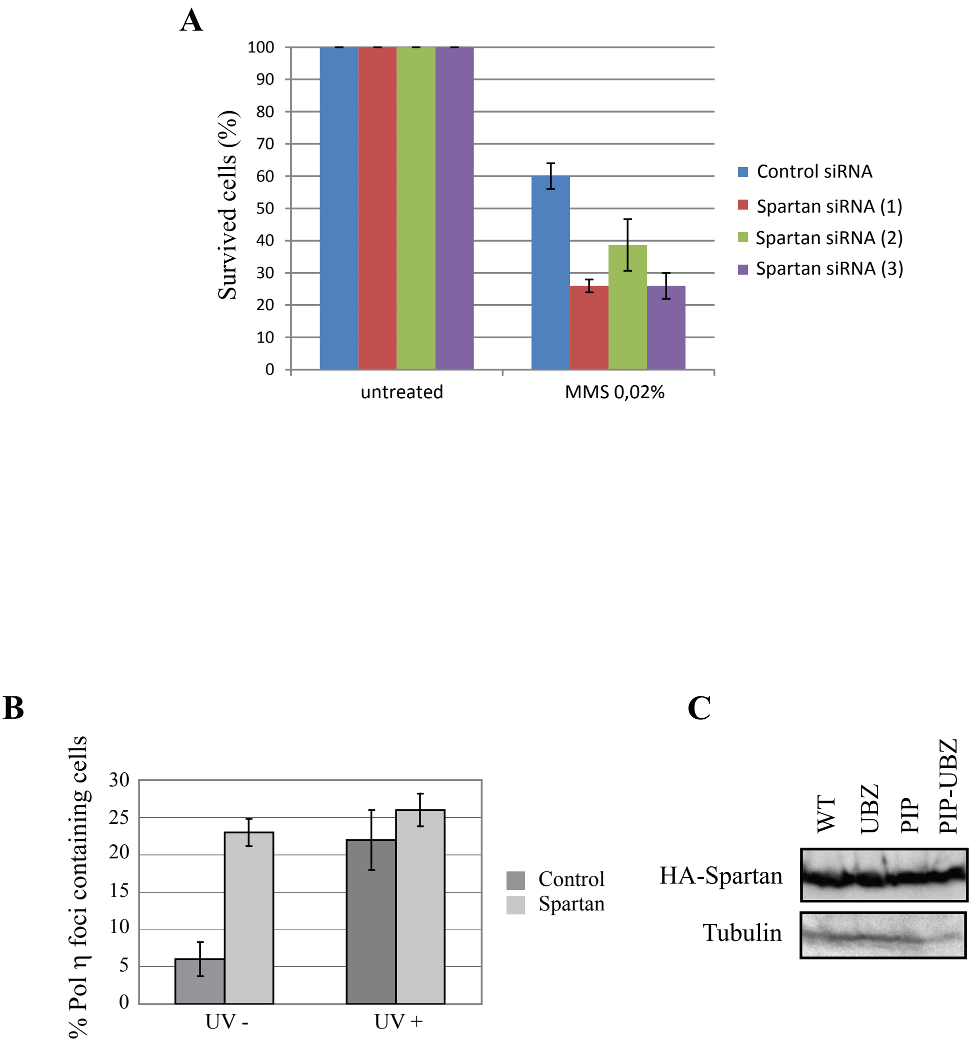

Supplementary Figure 3
